# Identification of the High Molecular Weight Isoform of Phostensin

**DOI:** 10.3390/ijms15011068

**Published:** 2014-01-15

**Authors:** Yu-Shan Lin, Hsien-Lu Huang, Wei-Ting Liu, Ta-Hsien Lin, Hsien-Bin Huang

**Affiliations:** 1 Department of Life Science and Institute of Molecular Biology, National Chung Cheng University, Chia-Yi 62102, Taiwan; E-Mails: lambdaphagecarol@yahoo.com.tw (Y.-S.L.); weiting0608@hotmail.com (W.-T.L.); 2 Department of Nutrition and Health Science, Fooyin University, Kaohsiung 83102, Taiwan; E-Mail: estrus@mail2000.com.tw; 3 Institute of Biochemistry and Molecular Biology, National Yang-Ming University, Taipei 11221, Taiwan; 4 Department of Medical Research & Education, Taipei Veterans General Hospital, Taipei 11217, Taiwan

**Keywords:** phostensin-α, phostensin-β, *KIAA1949*, protein phosphatase-1, actin-binding protein

## Abstract

Phostensin is encoded by *KIAA1949*. 5′-RACEanalysis has been used to identify the translation start site of phostensin mRNA, indicating that it encodes 165 amino acids with an apparent molecular weight of 26 kDa on SDS-PAGE. This low-molecular-weight phostensin is present in human peripheral blood mononuclear cells and many leukemic cell lines. Phostensin is a protein phosphatase-1(PP1) binding protein. It also contains one actin-binding motif at its *C*-terminal region and binds to the pointed ends of actin filaments, modulating actin dynamics. In the current study, a high-molecular-weight phostensin is identified by using immunoprecipitationin combination with a proteomic approach. This new species of phostensin is also encoded by *KIAA1949* and consists of 613 amino acids with an apparent molecular weight of 110 kDa on SDS-PAGE. The low-molecular-weight and high-molecular-weight phostensins were named as phostensin-α and phostensin-β, respectively. Although phostensin-α is the *C*-terminal region of phostensin-β, it is not degraded from phostensin-β. Phostensin-β is capable of associating with PP1 and actin filaments, and is present in many cell lines.

## 1. Introduction

Phostensin, a protein phosphatase-1 F-actin cytoskeleton targeting subunit, is encoded by *KIAA1949* [1–3]. The genomic location of phostensin is between the *HLA-C* and *HLA-E* gene clusters on human chromosome 6. Northern blot analysis indicated that phostensin mRNA was predominantly distributed in leukocytes and spleen. Phostensin is abundant in helper T-lymphocytes, cytotoxic T-lymphocytes, mature monocytes, macrophages, B-lymphocytes, granulocytes and natural killer cells [4].

The 5′-end of phostensin mRNA and the translation start site have been determined by using 5′-RACE analysis. Phostensin consists of 165 amino acids with one consensus protein phosphatase 1 (PP1)-binding motif located between residues 91 and 94. Phostensin was observed to associate with PP1 in yeast two-hybrid screens, co-immunoprecipitation and GST pull-down assays. A mutation at the PP1-binding motif of phostensin abolished PP1 binding [1]. Phostensin also contains one actin-binding motif located at residues 129–156 [5], accounting for the over-expressed phostensin co-localized with the actin cytoskeleton at the cell periphery in Madin-Darby canine kidney epithelial cells [1,5]. Phostensin binds to the pointed ends of actin filaments but not to actin monomers, sides of filaments, or barbed ends of filaments, retarding the elongation and depolymerization rates of gelsolin-actin seeds and reducing the rate of G-actin addition at the pointed ends [4,6].

The open reading frame of *KIAA1949* was predicted to encode a putative protein of 613 amino acids by the GENSCAN program [2,3]. However, this full-length transcript of *KIAA1949* gene was not observed by the early 5′-RACE analysis that only identified a shorter transcript encoding a smaller protein of 165 amino acids with an apparent molecular weight of 26 kDa on SDS-PAGE [1]. The sequence of this short-length protein was the *C*-terminal region of the predicted full-length protein. Examination of western blotting only identified the phostensin protein with 26 kDa on SDS-PAGE in crude extracts of human peripheral leukocytes [1,4]. In this study, we apply co-immunoprecipitationin combination with proteomic approach to analyze the crude protein extracts of Jurkat cells and identify a high-molecular-weight species of phostensin encoded by the full-length transcript of *KIAA1949* gene with an apparent molecular weight of 110 kDa on SDS-PAGE.

## 2. Results and Discussion

### 2.1. Identification of the High Molecular Weight of Phostensin

**Figure 1.**
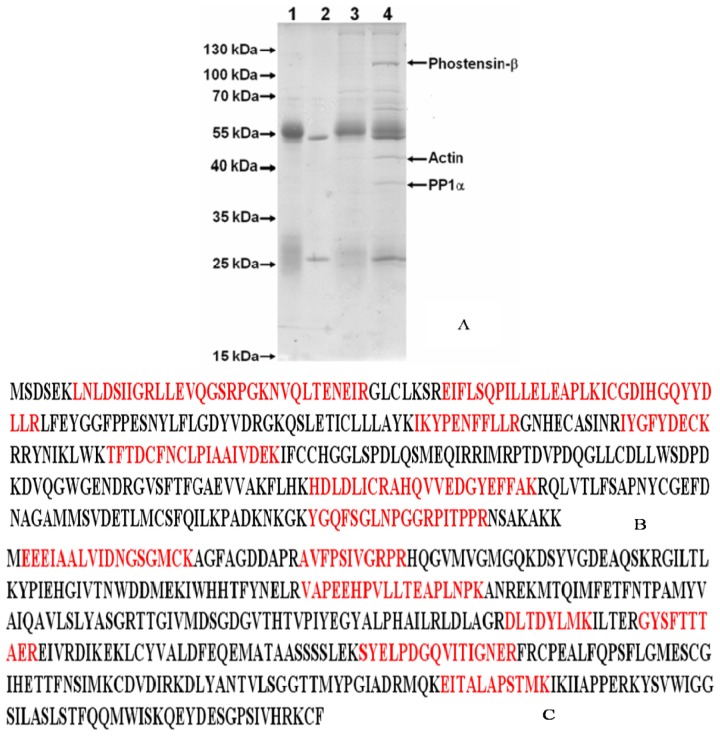
Identification of phostensin-binding protein by proteomic approaching. (**A**) SDS-PAGE analysis of the proteins co-immunoprecipitated by PT-2. The proteins indicated by the arrows on gel were identified by mass spectrometry and proteomics. The mobility for the low molecular weight of phostensin (26 kDa) identical to that of the light chain of IgG cannot be assigned in this study. Lane **1**: Rabbit anti-Mouse antibody (RAM); Lane **2**: Mouse anti-phostensin monoclonal antibody (PT-2); Lane **3**: Protein A beads + RAM + Jurkat cell lysate; Lane **4**: Protein A beads + RAM + Jurkat cell lysate + PT-2; (**B**) The amino acid sequence for PP1. The apparent molecular weight of PP1 on SDS-PAGE is 37 kDa (Figure 1**A**). Bolded peptides (red) were identified by mass spectrometry. Score: 611; and (**C**) The amino acid sequence of actin. The apparent molecular weight of actin on SDS-PAGE is 45 kDa (Figure 1**A**). Bolded peptides (red) were identified by mass spectrometry. Score: 259.

**Figure 2.**
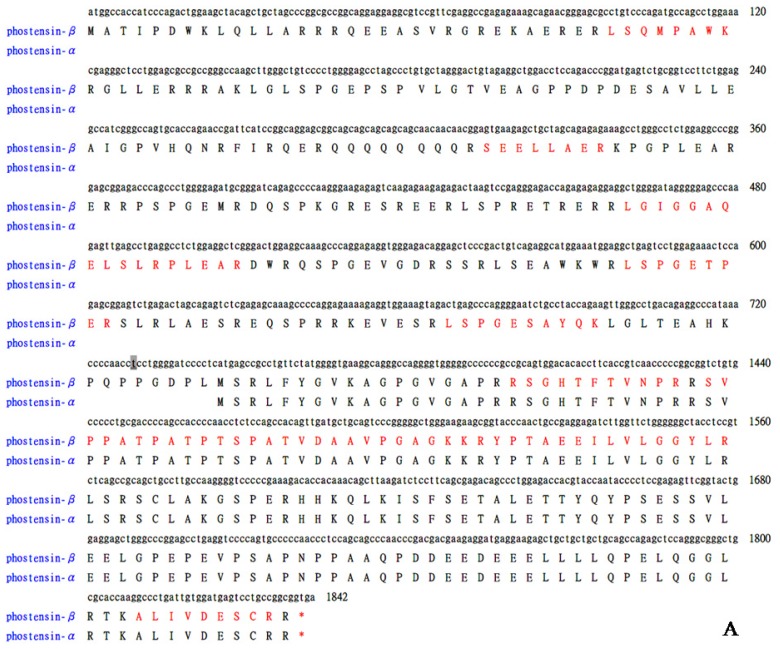

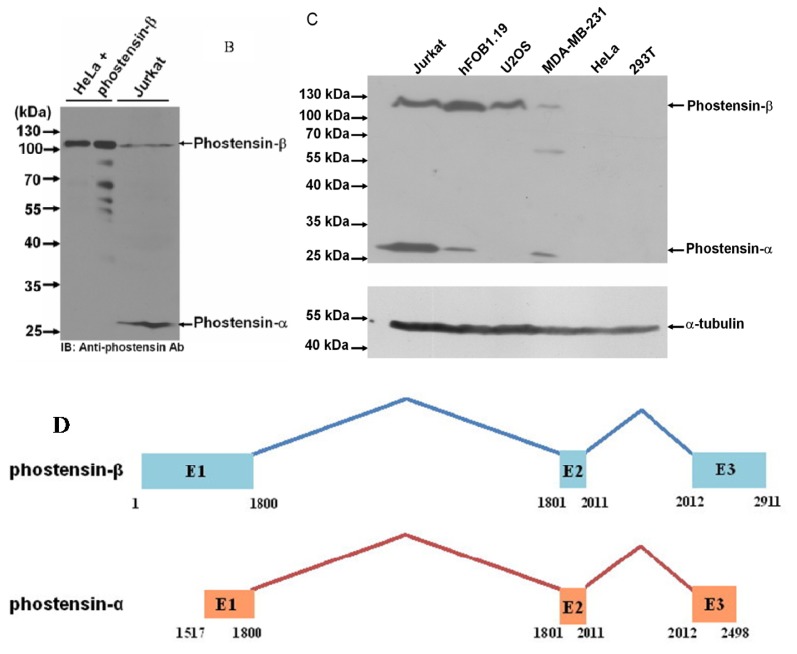
Identification of phostensin-β. (**A**) cDNA and encoded protein sequences of human phostensin-α and -β. The analyzed protein was isolated from the band with the apparent molecular weight of 110 kDa on SDS-PAGE of Figure 1A. Bolded peptides (red) were identified by mass spectrometry. Score: 738; (**B**) Western blotting analysis of phostensin-β overexpressed in HeLa cells. Phostensin-β was transiently overexpressed in HeLa cells. Cellular proteins were extracted by 1% SDS and ultrasonication. 5 and 10 μg of crude proteins extracted from HeLa and Jurkat cells, respectively, were analyzed by SDS-PAGE (10%), followed by western blotting using the PT2 monoclonal antibody; (**C**) Phostensin-β is present in many cell lines. Cellular proteins were extracted by the above-mentioned method. An aliquot (100 μg) of each extract was analyzed by western blotting using the PT2 monoclonal antibody; and (**D**) Phostensin-α and -β transcripts are transcribed from different starting sites. The *KIAA1949* transcript contains three exons (E1, E2 and E3). The transcriptional starting site of phostensin-α is identified by 5′-RACE and located within exon I. The exon III of phostensin-α transcript was not completely sequenced in our previously study [1].

Phostensin was highly expressed in Jurkat cells [4]. PT-2 is a monoclonal antibody that specifically recognizes the residues 89–124 of phostensin [4]. In order to examine the phostensin-binding proteins in cells and explore its biological functions, crude proteins extracted from the Jurkat cells were subjected to immunoprecipitation by PT-2. The PT-2-precipitated proteins were separated by SDS-PAGE. After stained with the Coomassie Brilliant Blue, each interesting band on gel was isolated (Figure 1A) and subjected to in-gel digestion. The resulting fragments were isolated and analyzed by mass spectrometry. The results indicated that PP1α and actin bind to phostensin, both of which can be co-immunoprecipitated by PT-2 (Figure 1A–C). A PT-2-precipitated protein with an apparent molecular weight of 110 kDa on gel was also analyzed and identified as a high-molecular-weight phostensin, named as phostensin-β (Figures 1A and 2A). The previously identified phostensin with the apparent weight of 26 kDa on SDS-PAGE is named as phostensin-α (Figure 2A) [1]. Phostensin-α is not identified in Figure 1A since its mobility is identical to the light chain of IgG on SDS-PAGE. However, the open reading frame of *KIAA1949* was predicted to encode a putative protein of 613 amino acids with the molecular weight of 67.0 kDa [2], much less than 110 kDa. Thus, it raised a question whether phostensin-β is encoded by a larger and unidentified open reading frame of *KIAA1949*. In order to examine this possibility, we overexpressed the transcript of *KIAA1949*, encoding 613 amino acids, in HeLa cells. The translated product was analyzed by western blotting. HeLa cell itself does not express phostensin-β (Figure 2C). After transfection with plasmids encoding *KIAA1949* with 613 amino acids, the resulting protein product appears at the apparent molecular weight of 110 kDa on gel. Its mobility is identical to phostensin-β of Jurkat cells (Figure 2B). Phostensin-β is present in many cell lines, such as Jurkat, hFOB 1.19, U2OS, MDA-MB-231 cells (Figure 2C), but not observed in HeLa and 293T cells. Phostensin-α and -β transcripts are transcribed from different starting sites (Figure 2D).

### 2.2. Phostensin-α Is not a Degraded Form of Phostensin-β

**Figure 3.**
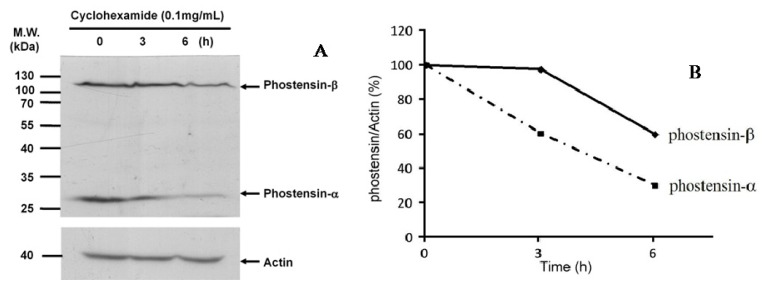
Phostensin-α is not derived from proteolytic degradation of phostensin-β. (**A**) Chase the degradation process of phostensin. Jurkat cells were cultured in RPMI supplemented with 10% FBS, cycloheximide (100 μg/mL) and 1% penicillin/streptomycin. Cells were harvested at the indicated time. Cellular proteins were extracted by 1% SDS. An aliquot (100 μg) of the extracted protein was separated by SDS-PAGE (10%) and analyzed by western blotting using PT2 monoclonal antibody and (**B**) Quantification of (**A**).

Figure 2C shows that both phostensin-α and -β are present in many cell lines. However, the amino acid sequence of phostensin-α is the *C*-terminal region of phostensin-β form (Figure 2A). It raises a question whether phostensin-α is derived from the proteolytic degradation of phostensin-β. To examine this possibility, we chased the degradation process of phostensin. Jurkat cells were cultured in the presence of cycloheximide and harvested at the indicated time. Crude proteins extracted from Jurkat cells were analyzed by western blotting (Figure 3A). Figure 3A,B indicates that phostensin-α and -β show different rates of proteolytic degradation. Phostensin-β is slightly degraded over the time, but the level of phostensin-α is not increased, suggesting that phostensin-α is not derived from phostensin-β through the proteolytic degradation. In addition, phostensin-α was not observed in HeLa cells that have over-expressed phostensin-β (Figure 2B). Thus, phostensin-α and -β are encoded by different length transcripts of *KIAA1949*. Indeed, northern blotting analysis revealed two major phostensin mRNA transcripts, ~1.5 and 4.2 kb in size, in many tissues [1].

### 2.3. Phostensin-β Binds to PP1 and F-Actin

**Figure 4.**
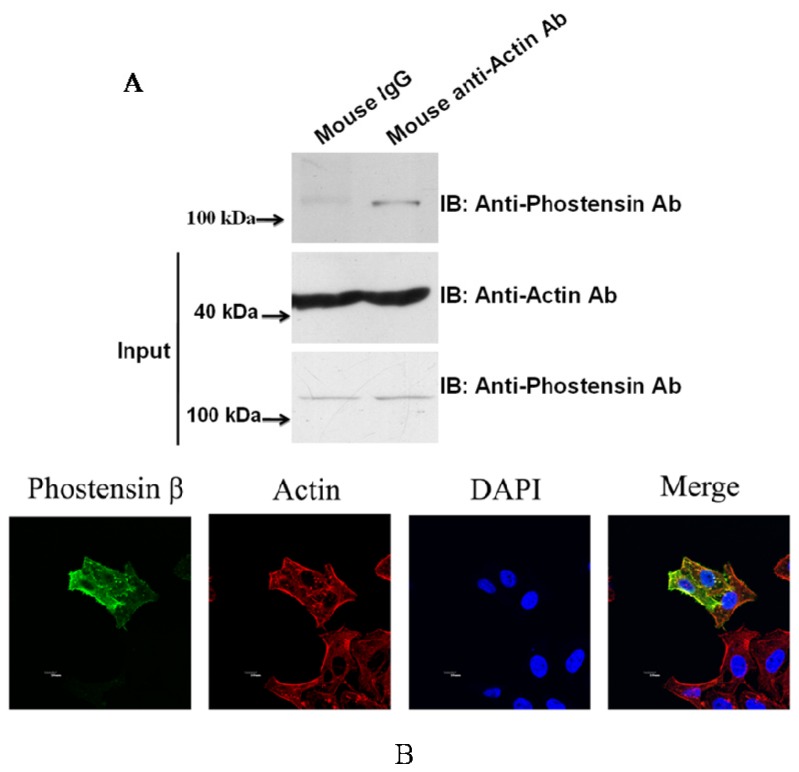
Phostensin-β binds to actin filament. (**A**) Endogenous phostensin-β is co-immunoprecipitated by anti-actin antibody. Crude proteins extracted from Jurkat cells were incubated with ant-actin antibody and then precipitated by protein G-Sepharose. After sedimentation, all components in the pellet were analyzed by western blotting using PT2 monoclonal antibody and (**B**) Co-localization of phostensin-β and F-actin in HeLa cells. HeLa cells were transiently transfected with pCMV-SPORT6 encoding phostensin-β. F-actin and phostensin-β were stained with rhodamine-phalloidin and Alexa-488-conjugated PT2 monoclonal antibody, respectively. Nucleus was stained with DAPI. Fluorescence for phostensin-β (green), F-actin (red) and nucleus (blue) is shown with the merged images. Scale bar: 10 μm.

**Figure 5.**
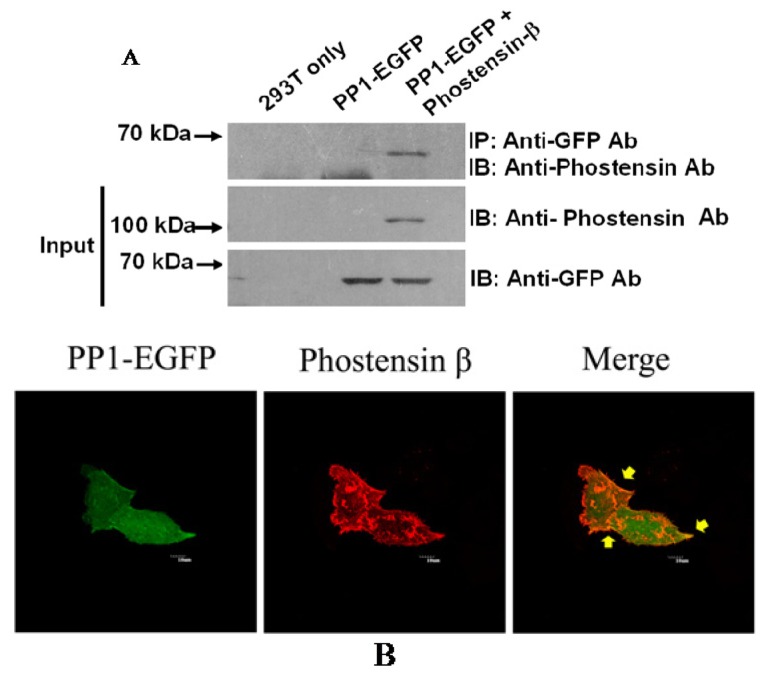
Phostensin-β binds to PP1. (**A**) PP1-EGFP is co-immunoprecipitated by anti-phostensin monoclonal antibody, PT2. 293T cells were transiently transfected with pPP1-EGFP and pCMV-SPORT6 encoding phostensin-β. Crude proteins extracted from transfected cells were incubated with the PT2 monoclonal antibody that pre-immobilized on protein G-Sepharose. After centrifugation, all components in the pellet were analyzed by western blotting using anti-EGFP antibody and (**B**) Co-localization of PP1 and phostensin-β in HeLa cells. Phostensin-β was pre-bound with PT2 monoclonal antibody and then stained with rhodamine-conjugated secondary antibody. Fluorescence for phostensin-β (red) and PP1-EGFP (green) is shown with the merged images. Arrows are used to indicate the complex of phostensin-β with PP1-EGFP. Scale bar, 10 μm.

Previous studies have demonstrated that phostensin-α binds to F-actin and PP1 [1,4–6]. Although PP1 and actin were precipitated by PT-2 monoclonal antibody from protein extracts of Jurkat cells, it may arise from interaction with phostensin-α instead of phostensin-β (Figure 1A). No evidence suggests that phostensin-β can also associate with F-actin and PP1. To examine this suggestion, crude proteins extracted from Jurkat cells were precipitated by anti-actin monoclonal antibody. The immunoprecipitated product was analyzed by western blotting using PT-2. Figure 4A shows that phostensin-β can be co-precipitated by actin. In addition, phostensin-β was transiently overexpressed in 293T cells which endogenous phostensin is un-detectable. Phostensin-β was firstly recognized by PT-2, followed by stain with Alexa-488-conjugated anti-mouse IgG antibodies. F-actin was stained with rhodamine-conjugated phalloidin. The result indicated that phostensin-β co-localizes with F-actin, concentrated at the peripheral cell junctions (Figure 4B). We also examined whether phostensin-β binds to PP1. Both PP1-EGFP and phostensin-β were transiently co-overexpressed in 293T cells. Crude proteins were extracted from transfected 293T cells and immunoprecipitaed by PT-2. The resulting product was analyzed by western blotting using anti-EGFP monoclonal antibody. Figure 5A shows that PP1-EGFP is co-immunoprecipitated by phostensin-β, suggesting that both proteins form a complex in cells. In addition, immunofluorescence microscopy also demonstrated that PP1-EGFP is co-localized with phostensin-β in transfected 293T cells (Figure 5B).

Although considerable biochemical information of phostensin, with respect to its interaction with actin filaments and PP1, has been revealed, the *in vivo* functions of phostensin remain to be elucidated. Immunohistochemical analyses of human thymus tissue demonstrated that phostensin is only present in the thymic medulla but not the thymic cortex, suggesting that phostensin is only expressed in mature Tcells [4]. In addition, promyelocytic leukemia cells (HL60) can be differentiated into granulocytes and monocytes, induced by retinoic acid and 1,25-dihydroxyvitamin D_3_, respectively. After differentiation, the expression of phostensin is significantly increased [4].

The genomic location of phostensin is between the *HLA-C* and *HLA-E* gene clusters on human chromosome 6 [2,3]. Cytogenetic aberration and loss of heterozygosity occurred on chromosome 6 in many cancers have been observed and tumorigenic potency of breast cancer cell lines can be suppressed by the introduction of a neo-tag chromosome 6 into the cancer cells [7–10]. Microarray studies have indicated that *KIAA1949* might be a potential breast cancer suppressor gene. *KIAA1949* is differentially expressed between MDA-MB-231, a tumorigenic and metastatic breast cancer cell line, and MDA/H6, the chromosome 6-mediated suppressed, non-tumorigenic, non-metastatic derivative cell line [7]. In addition, when compared with normal cell lines or tissues, the *KIAA1949* transcript was down-regulated in 86.7% of breast cancer lines and tumor tissues and was undetectable or significantly decreased in 10 breast cancer cell lines, including MDA-MB-231. Western blotting analysis in this study indicated that MDA-MB-231 cells express the low levels of phostensin-β and phostensin-α (Figure 2C). Phostensin binds to the pointed ends of actin filaments and retards their elongation and depolymerization rates [1,4–6]. Whether this function affects cancer cell invasion and metastasis needs to be further investigated. In addition, phostensincan target PP1, one of the major serine/threonine protein phosphatase that can reverse the action of protein kinases [11–13], to the pointed ends of actin filaments, modulating phosphorylation/dephosphorylation of the actin cytoskeleton network. This activity may also play a potential role in cancer cell invasion and metastasis.

A differential expression of phostensin-α and phostensin-β in human PBMCs was observed [4]. Human peripheral blood mononuclear cells (PBMCs) only express phostensin-α and no phostensin-β was found in protein crude extracts [4]. Jurkat cells and other leukemic cell lines can express both types of phostensin (data not shown). However, compared with human PBMCs, the expression of phostensin-α is significantly decreased in leukemic cell lines [4]. The significance of this differential expression in phostensin-α and -β between normal leukocytes and leukemic cell lines remains characterized.

## 3. Experimental Section

### 3.1. Materials

Dithiothreitol (DTT), Tris, Luria-Bertani (LB) broth, cycloheximide, amplicillin, imidazole, ATP, glucose, glucose oxidase, isopropyl β-d-1-thiogalactopyranoside (IPTG), phenylmethylsulfonyl fluoride (PMSF), and glycine were obtained from Sigma-Aldrich (St. Louis, MO, USA). Rhodamine-phalloidin and Alexa-488-phalloidin were purchased from Invitrogen (Carlsbad, CA, USA). The full-length of *KIAA1949* subcloned into the pCMV-SPORT6 plasmid by *Sal*I/*Not*I was ordered from Bioresource Collection and Research Center (Hsin-Chu, Taiwan).

### 3.2. Identification of Phostensin-β

Jurkat cells were harvested, and centrifuged at 1500× *g* for 5 min. After washing with PBS, the pelleted cells were ruptured by RIPA buffer [50 mM Tris-HCl, pH 7.4, 150 mM NaCl, 10 mM EDTA, 1% NP-40, 1% Triton X-100, 1 mM phenylmethylsulfonyl fluoride, aprotinin (0.2 U/mL), leupeptin (20 μg/mL)]. 6 mg of protein extractwere incubated with anti-phostensin monoclonal antibody (3 μg) for 60 min at 4 °C and then mixed with protein A-Sepharose (30 μL). After incubation at 4 °C for 4 h, the precipitates were washed four times with RIPA wash buffer (50 mM Tris-HCl, pH 7.4, 150 mM NaCl, 10 mM EDTA, 0.1% NP-40, 0.1% Triton X-100), and collected by centrifugation (760× *g*) at 4 °C for 2 min. The precipitated products were analyzed by SDS-PAGE and stained with Coomassie Brilliant Blue. The interested bands were subjected to analysis by liquid chromatography combined with tandem mass spectrometry (LC-MS/MS) (AB SCIEX Q-STAR, Framingham, MA, USA).

### 3.3. Cell Culture and Transfection

Jurkat cells were cultured in RPMI 1640 medium supplemented with 10% fetal calf serum. 293T and HeLa cells were cultured in Dulbecco’s modified Eagle’s medium (DMEM) (Hyclone, South Logan, UT, USA) supplemented with 10% fetal calf serum. MDA-MB-231 cells were cultured in Leibovitz’s L-15 medium (Hyclone) supplemented with 10% fetal calf serum. U2OS cells were cultured in McCOY’s 5a medium supplemented with 10% fetal calf serum. hFOB1.19 cells were cultured in a 1:1 mixture of Ham’s F12 Medium (Hyclone) with DMEM supplemented with 10% fetal calf serum. With the exception of MDA-MB-231 cells, all cells were cultured at 37 °C with 5% CO_2_. HeLaand 293T cells were transfected with the indicated amount of plasmid DNA using the Turbofect *in vitro* transfection reagent (Thermo Scientific, Hudson, NH, USA).

### 3.4. Chase the Degradation Process of Phostensin

Jurkat cells (2 × 10^5^ cells) were seeded into 6-cm petri dishes and maintained in PRMI supplemented with 10% FBS and 1% penicillin/streptomycin. Cells were incubated with cycloheximide (100 μg/mL) and were harvested at the indicated time by centrifugation at 1500× *g* for 5 min. Pelleted cells were resuspended in 0.1 mL 1% SDS and ruptured by ultrasonication. Aliquots of cell extracts were analyzed by SDS-PAGE and western blotting.

### 3.5. Immunoprecipitation

Jurkat cells were grown to confluence in 10-cm culture dishes and harvested by centrifugation at 1500× *g* for 5 min. After washing with PBS and centrifugation, the pelleted cells were ruptured by 1 mL of RIPA buffer. 500 μg of extracted proteins were subjected to immunoprecipitation by anti-actin monoclonal antibody pre-immobilized on protein G-Sepharose (25 μL). After incubation at 4 °C for 2 h, all components were washed with RIPA buffer for four times, and harvested by centrifugation (760× *g*) at 4 °C for 1.5 min, followed by analysis using western blotting using anti-phostensin monoclonal antibody, PT2. To examine PP1 interacted with phostensin-β, 293T cells pre-transfected with pPP1-EGFP and pCMV-SPORT6 encoding phostensin-β were cultured in DMEM supplemented with 10% FBS and 1% penicillin/streptomycin at 37 °C with 5% CO_2_ for 48 h. Cells were harvested by centrifugation and ruptured by 0.5 mL of RIPA buffer. Phostensin-β in the crude protein extract (300 μg) was immunoprecipitated by PT2, separated by SDS-PAGE and analyzed by western blotting using anti-EGFP antibody.

### 3.6. Immunocytochemistry

HeLa cells pre-transfected with pPP1-EGFP and pCMV-SPORT6 encoding phostensin-β were maintained in DMEM supplemented with 10% FBS and 1% penicillin/streptomycin at 37 °C with 5% CO_2_ for 16 h. For microscopy, the transfected cells were grown on slides. Cells were washed twice with PBS, fixed for 15 min in 4% paraformaldehyde, permeabilized for 15 min with 0.5% Triton X-100 in PBS, incubated with a 1:400 dilution of anti-phostensin monoclonal antibody PT2 for 60 min, and then incubated with a 1:400 dilution of Alexa-488-conjugated orrhodamine-conjugated secondary antibody (Life Technologies, Carlsbad, CA, USA). For staining actin filaments, a 1:400 dilution of rhodamine-conjugated phalloidin was used. Cells were analyzed with an Olympus FV1000 confocal microscope (Olympus Corporation, Tokyo, Japan).

## 4. Conclusions

We have demonstrated that *KIAA1949* can be transcribed into two transcripts. One encodes phostensin-α containing 165 amino acids and the other is translated to phostensin-β consisting of 613 amino acids. The apparent molecular weights for phostensin-α and phostensin-β on SDS-PAGE are 26 and 110 kDa, respectively, and are unexpectedly higher than those of theoretical molecular weight of 17,771 and 67,942 Da, respectively (Figures 1A and 2B). Although many cells can simultaneously express phostensin-α and -β (Figure 2C), human PBMC only express phostensin-α [4], while U2OS only expresses phostensin-β in western blotting analysis (Figure 2C). Phostensin-α is the *C*-terminal region of phostensin-β, but it is not protease-degraded form of phostensin-β (Figure 3). Like phostensin-α, phostensin-β can associate with F-actin and PP1, as evidenced by co-immunoprecipitation (Figures 1A, 4A and 5A) and immunofluorescence microscopy (Figures 4B and 5B). Whether the different types of phostensin can play different biological functions needs to be characterized.
